# Development of a patients’ satisfaction analysis system using machine learning and lexicon-based methods

**DOI:** 10.1186/s12913-023-09260-7

**Published:** 2023-03-23

**Authors:** Shiva Khaleghparast, Majid Maleki, Ghasem Hajianfar, Esmaeil Soumari, Mehrdad Oveisi, Hassan Maleki Golandouz, Feridoun Noohi, Maziar Gholampour dehaki, Reza Golpira, Saeideh Mazloomzadeh, Maedeh Arabian, Samira Kalayinia

**Affiliations:** 1 Cardiovascular Nursing Research Center, Rajaie Cardiovascular Medical and Research Center, Iran University of Medical Sciences, Tehran, Iran; 2 Rajaie Cardiovascular Medical and Research Center, Iran University of Medical Sciences, Tehran, Iran; 3 Department of Computer Science, University of British Columbia, Vancouver, BC Canada; 4 Department of Community Health Sciences, University of Manitoba, Winnipeg, Canada; 5 Cardiogenetic Research Center, Medical and Research Center, Rajaie Cardiovascular, University of Medical Sciences, Tehran, Iran

**Keywords:** Patients’ rights, Machine learning, Lexicon, Sentiment classification

## Abstract

**Background:**

Patients’ rights are integral to medical ethics. This study aimed to perform sentiment analysis and opinion mining on patients’ messages by a combination of lexicon-based and machine learning methods to identify positive or negative comments and to determine the different ward and staff names mentioned in patients’ messages.

**Methods:**

The level of satisfaction and observance of the rights of 250 service recipients of the hospital was evaluated through the related checklists by the evaluator. In total, 822 Persian messages, composed of 540 negative and 282 positive comments, were collected and labeled by the evaluator. Pre-processing was performed on the messages and followed by 2 feature vectors which were extracted from the messages, including the term frequency–inverse document frequency (TFIDF) vector and a combination of the multifeature (MF) (a lexicon-based method) and TFIDF (MF + TFIDF) vectors. Six feature selectors and 5 classifiers were used in this study. For the evaluations, 5-fold cross-validation with different metrics including area under the receiver operating characteristic curve (AUC), accuracy (ACC), F1 score, sensitivity (SEN), specificity (SPE) and Precision-Recall Curves (PRC) were reported. Message tag detection, which featured different hospital wards and identified staff names mentioned in the study patients’ messages, was implemented by the lexicon-based method.

**Results:**

The best classifier was Multinomial Naïve Bayes in combination with MF + TFIDF feature vector and SelectFromModel (SFM) feature selection (ACC = 0.89 ± 0.03, AUC = 0.87 ± 0.03, F1 = 0.92 ± 0.03, SEN = 0.93 ± 0.04, and SPE = 0.82 ± 0.02, PRC-AUC = 0.97). Two methods of assessment by the evaluator and artificial intelligence as well as survey systems were compared.

**Conclusion:**

Our results demonstrated that the lexicon-based method, in combination with machine learning classifiers, could extract sentiments in patients’ comments and classify them into positive and negative categories. We also developed an online survey system to analyze patients’ satisfaction in different wards and to remove conventional assessments by the evaluator.

**Supplementary Information:**

The online version contains supplementary material available at 10.1186/s12913-023-09260-7.

## Introduction

Patients’ rights were gradually recognized and expanded in industrial Europe and North America from the early 1964s until they spread to most European countries in the early 21st century. Patients’ needs are akin to individuals’ rights, and patients’ rights are the duties that a medical center owes to patients. To put it differently, patients’ rights are the expectations that patients have of healthcare providers [1]. The position of the patient in the healthcare system is a matter of great significance with a great impact on the observance of ethical principles [2]. The issue of patients’ rights has received a great deal of attention in the last 2 decades for reasons such as the vulnerability and need of patients on the one hand and the increasing attention of the international community on the other hand. The principle of observing the charter of patients’ rights in any society constitutes one of the most important ethical duties in the field of medical ethics, which has a long history in the medical world [3]. The purpose of such charters is to defend the rights of patients, to ensure the proper care of patients, to improve communication between patients and healthcare providers, and to enhance the quality of healthcare [4]. The nonobservance of patients’ rights leads to their dissatisfaction, which may manifest itself in several ways such as irritability, slow recovery, and lengthened hospitalization [5]. Patients’ satisfaction can be measured with the aid of the Hospital Consumer Assessment of Healthcare Providers and Systems (HCAHPS) questionnaire and other manual questionnaires; nevertheless, these methods are time-consuming and costly [6, 7]. To address the aforementioned shortcomings, researchers have devised sentiment analysis (SA) and opinion mining, which is capable of extracting individuals’ thoughts and opinions and classifying their polarity as positive, negative, and neutral [8]. In the medical field, the purpose of SA and opinion mining is to augment healthcare services rendered to patients by evaluating their consent or discontent expressed in their comments. The existing SA and opinion mining methods include machine learning (ML) [9], deep learning (DL) [10], and lexicon-based methods [11]. DL requires a vast amount of information. In the lexicon-based method, as the name implies, lexica are drawn upon. First, a relevant message is tokenized and then each word of the message is assigned a part-of-speech (POS) tag to determine their role in the sentence. Next, based on a prepared lexicon, the polarity of each word is determined. Finally, via polarity model, the overall feeling of the message is extracted [12]. A combination of lexicon-based and ML methods is employed to achieve a high accuracy (ACC) rate, and a hybrid selection method is adopted to prepare the feature vectors used in classifiers [13, 14].

There are several review studies [15–18] in SA and ML in the healthcare domain. Rodrigues et al. [19] developed SentiHealth-Cancer to classify patients’ messages to positive, negative, or neutral. In a similar study, Del-Arco et al. [20] used Corpus Of Patient Opinions in Spanish (COPOS) and ML to mine Spanish patient opinions. Greaves et al. [21] implement several ML methods to classify the opinion of patients’ experience in hospital by online comments. In another study, Gopalakrishnan and Ramaswamy [22] apply a neural network approach for opinion classification in drugs satisfaction. Alayba et al. [23] introduce the Arabic language dataset in health services by collecting from Twitter. They used several ML and neural network methods in opinion classification to positive and negative classes.

The main aim of this study was to perform SA and opinion mining on patients’ messages with the aid of a combination of lexicon-based and ML methods to identify positive and negative comments and to determine different ward and staff names mentioned in patients’ messages.

## Methods

**Fig. 1:**
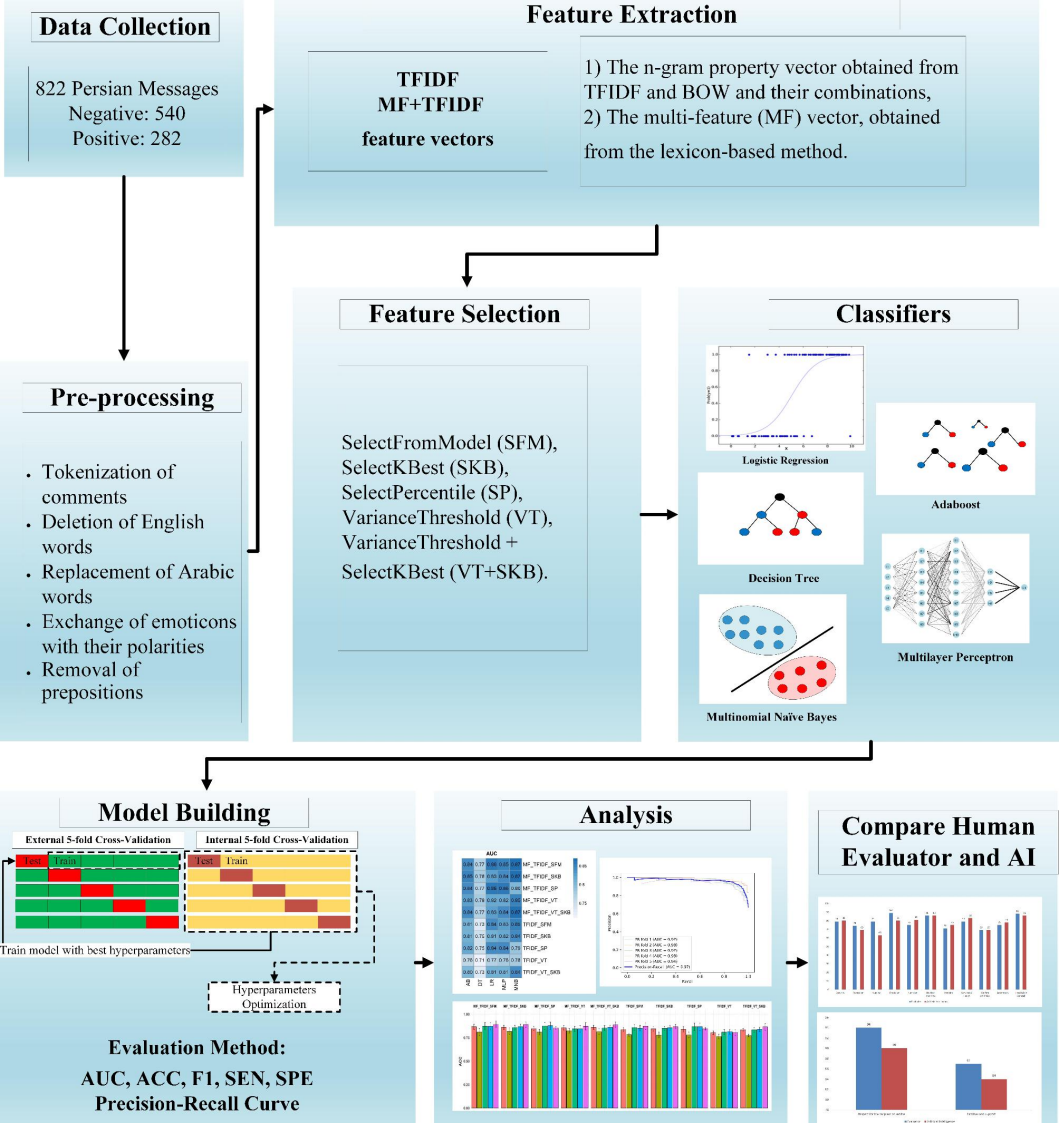
Flowchart of different steps of this study

Figure 1 shows flowchart of current study.

### Data collection

Patients’ messages were collected in the database of Rajaie Cardiovascular Medical and Research Center, affiliated with Iran University of Medical Sciences. All messages were reviewed by an educated analyzer and labelled to positive and negative classes due to patient comments. The text messages written in Persian were included if they contained enough words to accurately express the opinion or sentiment being expressed. Messages in any other languages and duplicated messages were excluded from this study. We also excluded neutral messages. In total, 822 messages, comprising 540 negative and 282 positive comments, were collected. Efforts were made to collect a wide range of data from the database with a view to affording the models a high degree of generalizability.

### Pre-processing

The data set was subjected to the necessary preprocessing, which included the tokenization of comments, the deletion of English words, the replacement of Arabic words with their Persian equivalents, the exchange of emoticons with their polarities, the substitution of a sequence of unnecessarily repetitive characters with 1 appropriate character (e.g. “veeeeery bad” with “very bad”, and the removal of prepositions.

### Feature extraction

The commonly used methods for extracting attributes include bag-of-words (BOW), term frequency–inverse document frequency (TFIDF), and Word2vec. In the present study, the following method was applied:

1. Each message was considered to consist of 1 or more sentences. At this point, each message was broken down into sentences.
2. After the sentences were specified, tokenization was performed on the messages so that the words of each sentence could be specified.
3. Phrases were distinguished in the sentences.
4. The TFIDF vector, used to extract attributes from text, was applied to determine the weight of each word in the phrases.
5. The words in each message were sorted in ascending order by their weight and the relevant words with the highest weight were selected by feature selection method.

The total feature vector was considered to comprise 2 parts: (1) the n-gram property vector obtained from TFIDF and BOW and their combinations, and (2) the multi-feature (MF) vector, obtained from the lexicon-based method. Information was obtained using (a) a list of different words with their polarity (i.e. positive and negative), (b) a booster word list of emotions, (c) a list of words expressing negative feelings (e.g., “not great”), and (d) a list of emoticons, each with 2 different polarities (i.e., positive and negative). The lists were prepared manually and were continually updated. The MF vector was considered to encompass the number of positive words, negative words, negative signs, neutralizing signs, emoticons, words longer than 2 letters, and punctuation signs (i.e., symbols such as ? and !).

TFIDF and MF + TFIDF feature vectors were utilized for further analysis, and NLTK and HAZM libraries were used for natural language processing to extract features.

### Feature selection

Our datasheet input was a text. In this type of information, the dimensions of feature vectors are usually high, undermining the function of ML algorithms and leading to overfitting. Accordingly, it is essential to select algorithms for more meaningful features.

The feature selectors applied in this study were SelectFromModel (SFM), SelectKBest (SKB), SelectPercentile (SP), VarianceThreshold (VT), and VarianceThreshold + SelectKBest (VT + SKB). Each of these feature selectors, based on a function, tries to select the best features from the feature vector and renders them as inputs to the classifiers.

### Machine learning classifiers

AdaBoost (AB), Decision Tree (DT), Logistic Regression (LR), Multilayer Perceptron (MLP), and Multinomial Naïve Bayes (MNB) comprised the classifiers employed in the current study.

### Model building and evaluation method

In the first step, we implemented external 5-fold cross-validation to split the dataset into training and testing sets each fold stratified by message labels. In each fold, we optimized hyperparameters of the classifiers with grid search and internal 5-fold cross-validation. Detail of these hyperparameters and other parameters of the classifiers were shown in Supplemental Table S1. Next, we trained the classifiers with optimal hyperparameters and made a model with the Pipeline function in which feature vectors, feature selectors, and classifiers are the inputs of this function. This process was repeated 5 times for each model.

In this study, we defined positive message to 0 and negative message to 1, and these metrics were calculated:

True Positive (TP): Patients’ message had negative label and the model classified it correctly.

True Negative (TN): Patients’ message had positive label and the model classified it correctly.

False Positive (FP): Patients’ message had positive label and the model classified it to negative.

False Negative (FN): Patients’ message had negative label and the model classified it to positive.

$$\text{A}\text{c}\text{c}\text{u}\text{r}\text{a}\text{c}\text{y}=\frac{\text{T}\text{P}+\text{T}\text{N}}{\text{T}\text{P}+\text{T}\text{N}+\text{F}\text{P}+\text{F}\text{N}}$$

$$\text{F}1 \text{S}\text{c}\text{o}\text{r}\text{e} =\frac{2\times \text{T}\text{P}}{2\times \text{T}\text{N}+\text{F}\text{P}+\text{F}\text{N}}$$

$$\text{S}\text{e}\text{n}\text{s}\text{i}\text{t}\text{i}\text{v}\text{i}\text{t}\text{y} =\frac{\text{T}\text{P}}{\text{T}\text{P}+\text{F}\text{N}}$$

$$\text{S}\text{p}\text{e}\text{c}\text{i}\text{f}\text{i}\text{c}\text{i}\text{t}\text{y} =\frac{\text{T}\text{P}}{\text{T}\text{N}+\text{F}\text{P}}$$

Mean and standard deviation metrics, including area under the receiver operating characteristic curve (AUC), ACC, F1 score, sensitivity (SEN), and specificity (SPE), were reported. We also prepared Precision-Recall Curves (PRC) for best model.

### Message tag detection

The purpose of this part was to determine to which ward or wards of the hospital patients’ messages referred. For instance, patients may express satisfaction or dissatisfaction with the nursing department, the pharmacy, or the laboratory in their comments and the objective is to identify the ward. ML and lexicon-based methods can serve this purpose; nonetheless, the dearth of available data prompted us to utilize only the latter method in this study. Consequently, the hospital wards (n = 53) were listed and a list of tags was prepared for each ward. If any of the words on the list were mentioned in a patient’s message, the comment was linked to the relevant ward. For example, the list of tags for the finance department included “finance”, “insurance”, “accounting department staff”, and “discharge staff”. This study also drew upon natural language processing (NLP) techniques vis-à-vis the Persian language given the possibility of a patient referring to a certain ward using various words. By way of example, a patient may refer to the nursing department by using words such as “nurse”, “nurses”, and “nursing staff”.

### Identifying staff names

The purpose of this section was to identify the staff names mentioned in the patients’ messages. Approximately, 2000 staff members with first name, surname prefixes (e.g. Mr., Miss., Dr. and etc.) were registered on the books of the hospital where the present study was conducted. Naturally, there were instances of similarity in terms of first name, surname, or both. Some staff members had surnames with affixes, giving rise to situations in which patients failed to mention the prefix or suffix. All these obstacles had to be cleared with the aid of proper filtering. For example, if a patient’s message bore the name “Dr. Ebrahimi”, with the surname “Ebrahimi” shared by 20 staff members, our algorithm returned only the items featuring the title “Dr.”. The algorithm used was based on n-gram search, comprising 1-gram, 2-gram, 3-gram, and 4-gram matches. A list of hospital staff names was provided to us, and the names were searched and returned based on the list.

### Web application service

**Fig. 2:**
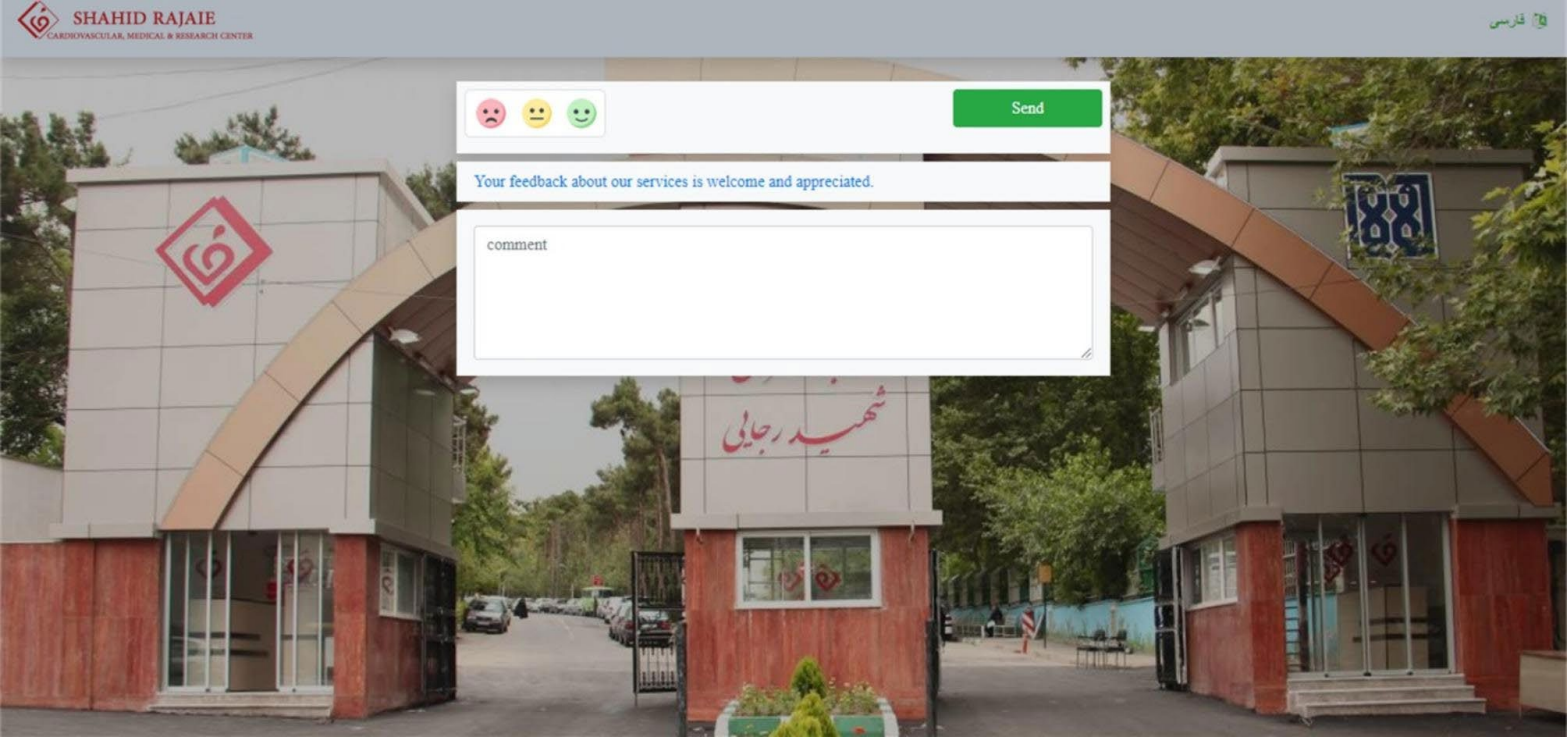
The image depicts the landing page form for receiving comments and sending them to API.

For the collection of user comments, a PHP website was designed on a Linux server (http://nazar.rhc.ac.ir/). Additionally, messages were sent in JSON format to the web API. (The Django framework was used). Ward tags and staff names were extracted from the message, and the best model was applied to the comment and the sentiment thereof (positive or negative). The probability prediction was sent back to the server in JSON format. Figure 2 depicts the webpage form for the collection of comments.

### Assessment by the evaluator

The level of satisfaction and observance of the rights of 250 service recipients of the hospital was evaluated through the related checklists using the method of simple random sampling for a period of six months. The inclusion criteria of the target patients were based on their willingness to communicate with the assessor and respond to the checklist questions. Accordingly, incomplete checklists were excluded from the study. To collect data, the assessor used interviews, observation, and review of documents. Furthermore, the checklists were completed by patients or a trained assessor based on the nature of the criterion. The research instruments included two sections for this part of the study. The first section was the standard checklist of the observance of service recipients’ rights based on the criteria of the comprehensive national accreditation guide for Iranian hospitals. This checklist was used according to the notification letter No. 13,625/130/D/97 dated 2018/10/16 given by the vice chancellor of the treatment deputy of the university. The above instrument consisted of two subcategories of respecting recipients’ rights, facilities and supports. The questions were evaluated in a Likert scale with a score of 1 (for the answer yes), 0.5 (for the answer to some extent), and 0 (for the answer no). The obtainable scores were between 0 and 49 for the subscale of respecting service recipients’ rights, between 0 and 122 for the subscale of facilities and supports. The total score of observing the service recipients’ rights was between 0 and 171 as well.

The second section was the validated checklist of measuring patients’ satisfaction with the code of FM 28 − 02, edition of July 29th, 2017. It included 67 statements for assessing the level of satisfaction with the services provided in various departments. Following entering the data in EXCEL software and analyzing the information, results from the scores were reported as the percentage of the observance of service recipients’ rights and also the percentage of patients’ satisfaction with the services received.

## Results

**Fig. 3:**
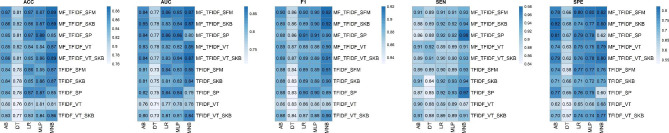
The image presents the performance of the different feature selectors across the classifiers on accuracy (ACC), area under the receiver operating characteristic curve (AUC), F1 score, sensitivity (SEN), and specificity (SPE) metrics

**Fig. 4:**
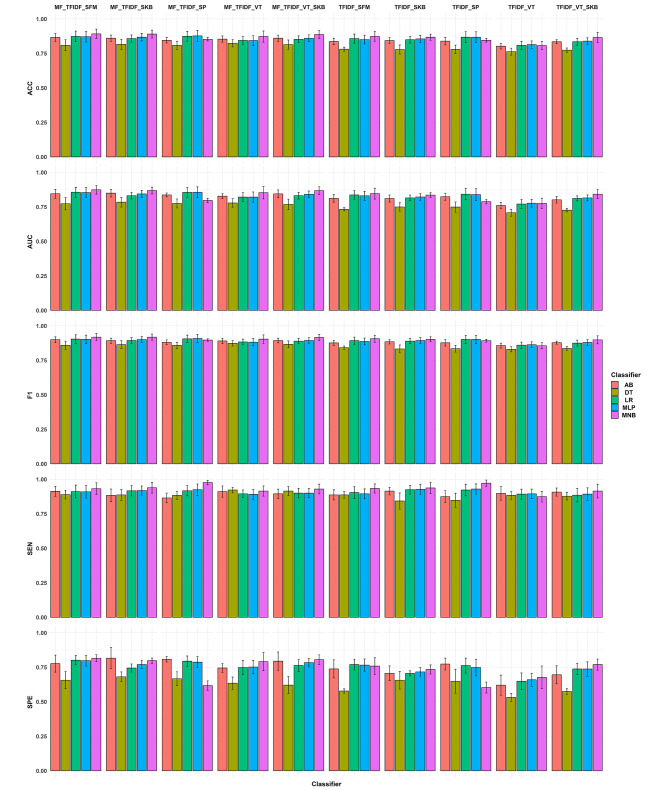
The image presents the mean and standard deviation (SD) of the different models with accuracy (ACC), area under the receiver operating characteristic curve (AUC), F1 score, sensitivity (SEN), and specificity (SPE) metrics

**Fig. 5:**
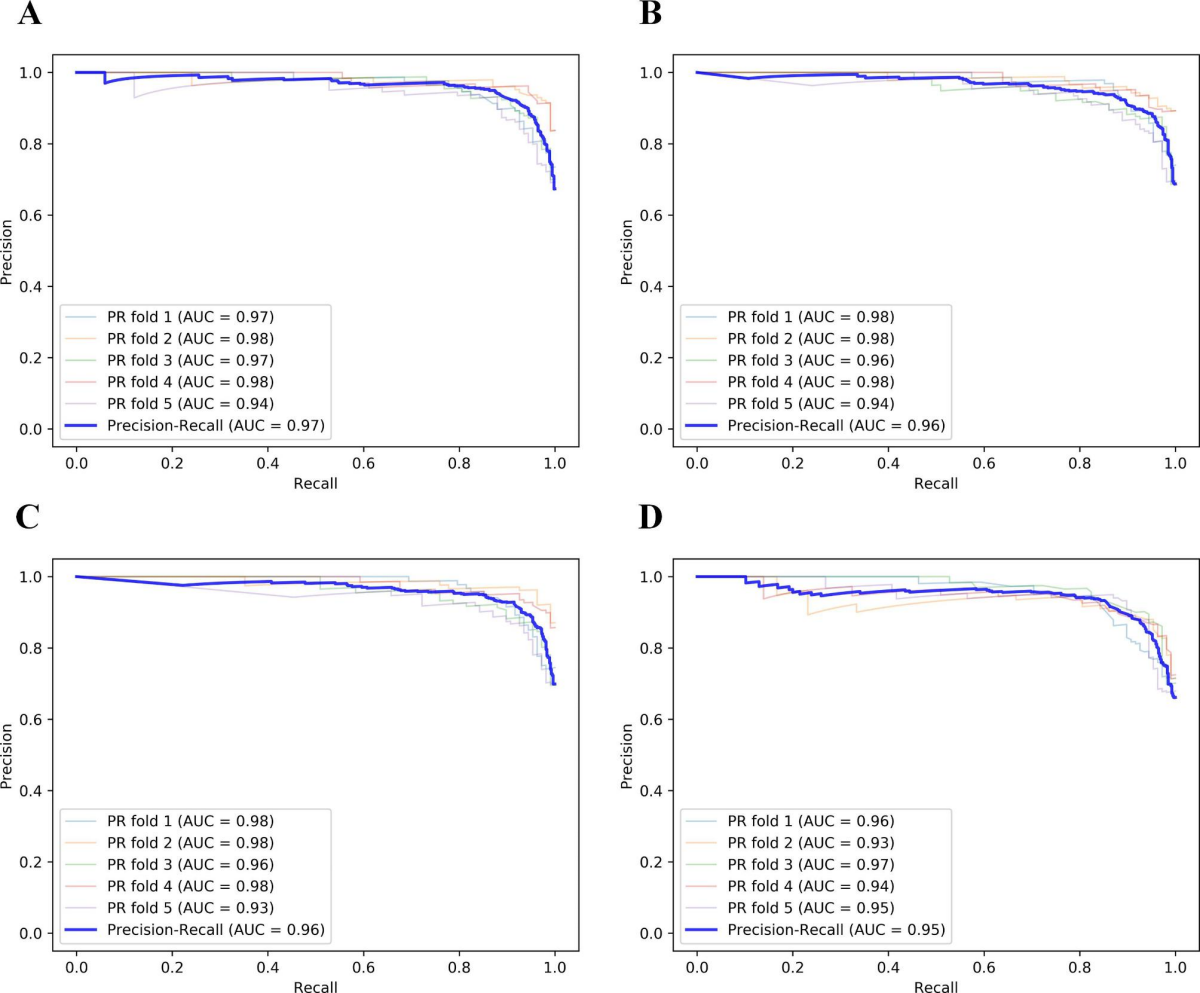
Precision-Recall curve of MNB in combination with MF + TFIDF and SFM (A), SKB (B), and VT + SKB (C), AB + MF + TFIDF + SKB (D). AB: AdaBoost, MNB: Multinomial Naïve Bayes (MNB), TFIDF: term frequency–inverse document frequency, MF: multi-feature, SFM: SelectFromModel, SKB: SelectKBest

The results of our different feature selectors across the classifiers with their respective ACC, AUC, F1 score, SEN, and SPE metrics performance are presented in Fig. 3. Additionally, Fig. 4; Table 1 show the mean and standard deviation of the different models employed in this study. PRCs of the best model were shown in Fig. 5. The best classifier was MNB in combination with MF + TFIDF and SFM (ACC = 0.89 ± 0.03, AUC = 0.87 ± 0.03, F1 = 0.92 ± 0.03, SEN = 0.93 ± 0.04, and SPE = 0.82 ± 0.02, PRC-AUC = 0.97 in Fig. 5-A), SKB (ACC = 0.89 ± 0.03, AUC = 0.87 ± 0.02, F1 = 0.92 ± 0.02, SEN = 0.94 ± 0.04, and SPE = 0.80 ± 0.02, PRC-AUC = 0.96 in Fig. 5-B), and VT + SKB (ACC = 0.89 ± 0.03, AUC = 0.87 ± 0.03, F1 = 0.91 ± 0.02, SEN = 0.93 ± 0.04, and SPE = 0.80 ± 0.04, PRC-AUC = 0.96 in Fig. 5-C), followed by AB + MF + TFIDF + SKB (ACC = 0.86 ± 0.02, AUC = 0.85 ± 0.03, F1 = 0.89 ± 0.02, SEN = 0.88 ± 0.05, and SPE = 0.82 ± 0.08, PRC-AUC = 0.95 in Fig. 5-D ).

**Table 1:** Mean and SD of different models with accuracy (ACC), area under the ROC curve (AUC), F1 score, sensitivity (SEN), and specificity (SPE) metrics

Metric		AB	DT	LR	MLP	MNB
ACC	MF_TFIDF_SFM	0.87 ± 0.03	0.81 ± 0.04	0.87 ± 0.04	0.87 ± 0.04	0.89 ± 0.03
	MF_TFIDF_SKB	0.86 ± 0.02	0.82 ± 0.04	0.86 ± 0.03	0.87 ± 0.03	0.89 ± 0.03
	MF_TFIDF_SP	0.85 ± 0.02	0.81 ± 0.03	0.87 ± 0.04	0.88 ± 0.04	0.85 ± 0.01
	MF_TFIDF_VT	0.85 ± 0.02	0.82 ± 0.03	0.84 ± 0.03	0.84 ± 0.03	0.87 ± 0.04
	MF_TFIDF_VT_SKB	0.86 ± 0.02	0.81 ± 0.03	0.85 ± 0.02	0.86 ± 0.02	0.89 ± 0.03
	TFIDF_SFM	0.84 ± 0.02	0.78 ± 0.01	0.86 ± 0.03	0.85 ± 0.03	0.87 ± 0.03
	TFIDF_SKB	0.84 ± 0.02	0.78 ± 0.03	0.85 ± 0.02	0.86 ± 0.03	0.87 ± 0.02
	TFIDF_SP	0.84 ± 0.03	0.78 ± 0.03	0.87 ± 0.04	0.87 ± 0.04	0.85 ± 0.01
	TFIDF_VT	0.8 ± 0.02	0.76 ± 0.03	0.81 ± 0.03	0.81 ± 0.03	0.81 ± 0.03
	TFIDF_VT_SKB	0.83 ± 0.01	0.77 ± 0.02	0.83 ± 0.02	0.84 ± 0.02	0.86 ± 0.04
AUC	MF_TFIDF_SFM	0.84 ± 0.03	0.77 ± 0.04	0.86 ± 0.04	0.85 ± 0.04	0.87 ± 0.03
	MF_TFIDF_SKB	0.85 ± 0.03	0.78 ± 0.04	0.83 ± 0.02	0.84 ± 0.03	0.87 ± 0.02
	MF_TFIDF_SP	0.84 ± 0.01	0.77 ± 0.03	0.86 ± 0.04	0.86 ± 0.04	0.8 ± 0.02
	MF_TFIDF_VT	0.83 ± 0.02	0.78 ± 0.03	0.82 ± 0.03	0.82 ± 0.04	0.85 ± 0.04
	MF_TFIDF_VT_SKB	0.84 ± 0.03	0.77 ± 0.04	0.83 ± 0.02	0.84 ± 0.02	0.87 ± 0.03
	TFIDF_SFM	0.81 ± 0.03	0.73 ± 0.01	0.84 ± 0.03	0.83 ± 0.03	0.85 ± 0.04
	TFIDF_SKB	0.81 ± 0.03	0.75 ± 0.03	0.81 ± 0.02	0.82 ± 0.02	0.84 ± 0.02
	TFIDF_SP	0.82 ± 0.03	0.75 ± 0.04	0.84 ± 0.04	0.84 ± 0.04	0.79 ± 0.02
	TFIDF_VT	0.76 ± 0.02	0.71 ± 0.03	0.77 ± 0.03	0.78 ± 0.03	0.78 ± 0.04
	TFIDF_VT_SKB	0.8 ± 0.02	0.73 ± 0.01	0.81 ± 0.02	0.81 ± 0.02	0.84 ± 0.03
F1	MF_TFIDF_SFM	0.9 ± 0.02	0.86 ± 0.03	0.9 ± 0.03	0.9 ± 0.03	0.92 ± 0.03
	MF_TFIDF_SKB	0.89 ± 0.02	0.86 ± 0.03	0.89 ± 0.02	0.9 ± 0.02	0.92 ± 0.02
	MF_TFIDF_SP	0.88 ± 0.02	0.86 ± 0.02	0.91 ± 0.03	0.91 ± 0.03	0.9 ± 0.01
	MF_TFIDF_VT	0.89 ± 0.02	0.87 ± 0.02	0.88 ± 0.02	0.88 ± 0.03	0.9 ± 0.03
	MF_TFIDF_VT_SKB	0.89 ± 0.02	0.87 ± 0.02	0.89 ± 0.02	0.89 ± 0.02	0.91 ± 0.02
	TFIDF_SFM	0.88 ± 0.02	0.84 ± 0.01	0.89 ± 0.03	0.89 ± 0.03	0.91 ± 0.03
	TFIDF_SKB	0.88 ± 0.02	0.83 ± 0.03	0.89 ± 0.02	0.89 ± 0.02	0.9 ± 0.02
	TFIDF_SP	0.88 ± 0.02	0.83 ± 0.03	0.9 ± 0.03	0.9 ± 0.03	0.89 ± 0.01
	TFIDF_VT	0.86 ± 0.02	0.83 ± 0.02	0.86 ± 0.02	0.86 ± 0.02	0.86 ± 0.02
	TFIDF_VT_SKB	0.88 ± 0.01	0.84 ± 0.01	0.87 ± 0.02	0.88 ± 0.02	0.9 ± 0.03
SEN	MF_TFIDF_SFM	0.91 ± 0.04	0.89 ± 0.03	0.91 ± 0.05	0.91 ± 0.05	0.93 ± 0.04
	MF_TFIDF_SKB	0.88 ± 0.05	0.89 ± 0.04	0.92 ± 0.04	0.92 ± 0.03	0.94 ± 0.04
	MF_TFIDF_SP	0.86 ± 0.04	0.88 ± 0.03	0.92 ± 0.04	0.92 ± 0.04	0.98 ± 0.02
	MF_TFIDF_VT	0.91 ± 0.04	0.92 ± 0.02	0.89 ± 0.03	0.89 ± 0.04	0.91 ± 0.04
	MF_TFIDF_VT_SKB	0.89 ± 0.03	0.91 ± 0.03	0.9 ± 0.03	0.9 ± 0.03	0.93 ± 0.04
	TFIDF_SFM	0.89 ± 0.04	0.89 ± 0.02	0.9 ± 0.04	0.89 ± 0.04	0.93 ± 0.03
	TFIDF_SKB	0.91 ± 0.03	0.84 ± 0.06	0.92 ± 0.03	0.93 ± 0.04	0.94 ± 0.04
	TFIDF_SP	0.87 ± 0.04	0.85 ± 0.05	0.92 ± 0.04	0.93 ± 0.04	0.97 ± 0.02
	TFIDF_VT	0.9 ± 0.05	0.88 ± 0.03	0.89 ± 0.04	0.89 ± 0.03	0.87 ± 0.04
	TFIDF_VT_SKB	0.91 ± 0.03	0.88 ± 0.03	0.89 ± 0.05	0.89 ± 0.05	0.91 ± 0.05
SPE	MF_TFIDF_SFM	0.78 ± 0.06	0.66 ± 0.06	0.8 ± 0.03	0.8 ± 0.04	0.82 ± 0.02
	MF_TFIDF_SKB	0.82 ± 0.08	0.68 ± 0.04	0.74 ± 0.03	0.77 ± 0.03	0.8 ± 0.02
	MF_TFIDF_SP	0.81 ± 0.02	0.67 ± 0.05	0.79 ± 0.04	0.79 ± 0.04	0.62 ± 0.03
	MF_TFIDF_VT	0.74 ± 0.03	0.63 ± 0.04	0.75 ± 0.05	0.75 ± 0.05	0.79 ± 0.07
	MF_TFIDF_VT_SKB	0.79 ± 0.07	0.62 ± 0.06	0.76 ± 0.04	0.78 ± 0.03	0.8 ± 0.04
	TFIDF_SFM	0.74 ± 0.07	0.58 ± 0.02	0.77 ± 0.04	0.77 ± 0.04	0.76 ± 0.06
	TFIDF_SKB	0.71 ± 0.05	0.66 ± 0.06	0.71 ± 0.02	0.72 ± 0.03	0.73 ± 0.03
	TFIDF_SP	0.77 ± 0.04	0.65 ± 0.09	0.76 ± 0.05	0.75 ± 0.06	0.6 ± 0.04
	TFIDF_VT	0.62 ± 0.07	0.53 ± 0.03	0.65 ± 0.06	0.66 ± 0.05	0.68 ± 0.08
	TFIDF_VT_SKB	0.7 ± 0.07	0.57 ± 0.02	0.74 ± 0.04	0.74 ± 0.05	0.77 ± 0.04

**Fig. 6:**
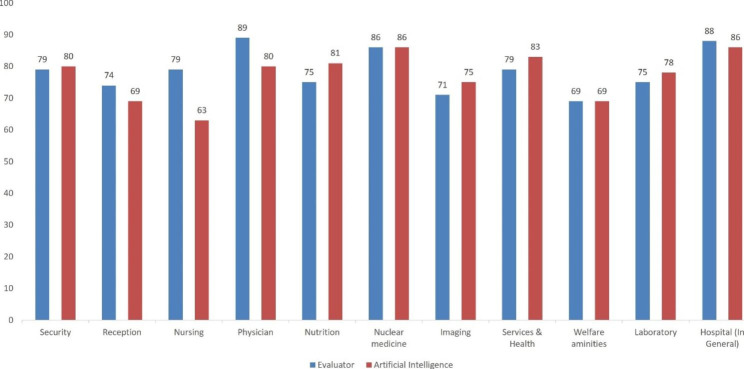
The image provides the comparison of the percentage of patients’ satisfaction in Rajaie Cardiovascular Medical and Research Center via 2 methods of assessment by the evaluator and the survey system

**Fig. 7:**
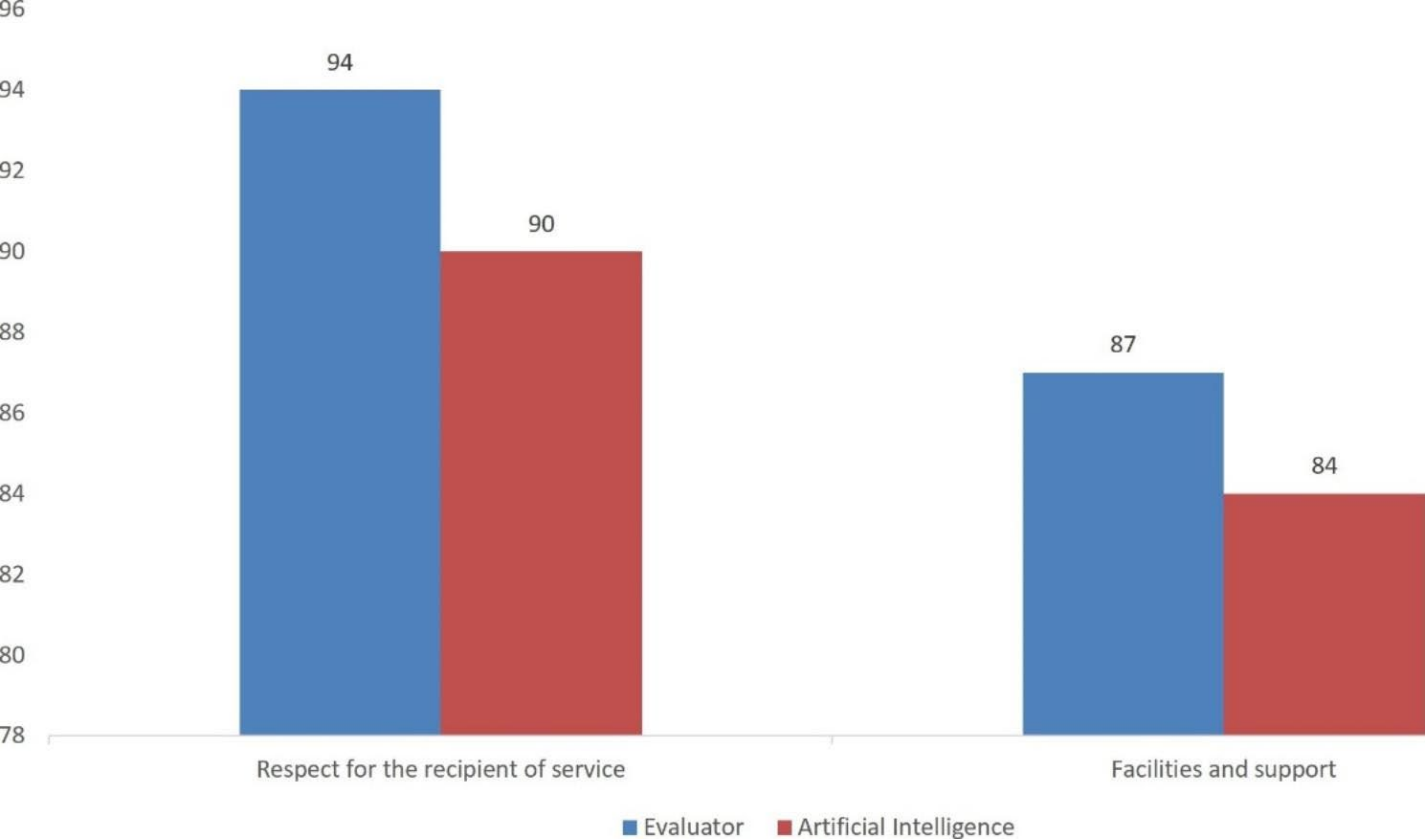
The image shows the comparison of the percentage of observance of service recipients’ rights in 2 methods of assessment by the evaluator and the survey system

The percentage of observance of patients’ rights in Rajaie Cardiovascular Medical and Research Center was calculated using the center’s electronic survey system. Two methods of assessment by the evaluator and artificial intelligence were used to determine and compare the satisfaction percentage of the patients (Fig. 6), and 2 methods of assessment by the evaluator and survey systems were utilized to determine and compare the percentage of observance of the patients’ rights in the hospital (Fig. 7).

## Discussion

In this study, we employed 2 feature extraction methods, in conjunction with different feature selectors and ML classifiers, for SA and opinion mining on 822 Persian comments made by patients at Rajaie Cardiovascular Medical and Research Center. The results of our study indicated that based on the methods of assessment by the evaluator, artificial intelligence, and survey systems, the satisfaction percentage of the patients and the observance percentage of service recipients’ rights were acceptable. A descriptive cross-sectional study by Fattahi et al. (2010) showed that from the patients’ point of view, the observance of patients’ rights by the medical staff was relatively acceptable. The observance of patients’ rights can help improve the quality of care and trust in medical centers.

To the best of our knowledge, our study is the first SA (opinion mining) and ML in the Persian language to classify messages into positive and negative and determine different hospital wards and staff names in patients’ comments. Several studies in other languages have also used SA and ML to classify patients’ comments. Asghar et al. [24] made use of a hybrid method (i.e., bootstrapping and lexicon-based SA to classify more than 26 000 comments as positive, negative, and neutral and achieved an ACC rate of 0.78. In their investigation, Del-Arco et al. [20] applied a corpus-based model, in combination with Support Vector Machine (SVM), to 743 comments and attained an ACC rate of 0.87. ElMessiry et al. [25] used a hybrid method that combined the classifiers of Linguist Inquiry Word Count (LIWC) and Naïve Bayes (NB) to categorize 7400 patients’ complaints and reported an ACC rate of 0.84. In a study by Rahim et al. [26], positive expressions made in the hospital and Facebook reviews in Malaysia were assessed by ML. The investigators classified 1852 reviews with the aid of the SVM classifier (ACC = 0.87 and recall = 0.93). In a similar work, Greaves et al. used SA and ML to capture patients’ experiences from online comments. They analyzed 6412 online comments and reported that the NB classifier had the best performance in terms of the prediction of the overall rating of the hospital (AUC = 0.94 and ACC = 0.88). Yenkikar et al. study investigated Twitter SA by using cascade feature selection (CSF) and Best Trained Ensemble (BTE) classified tweets in six public Twitter sentiment datasets. BTE had better performance than other classifiers (F-score range from 0.72 to 0.95 in different datasets [27]. Du et al. developed an ML model for the classification of HPV vaccination sentiment by 6000 public opinions on Twitter. Micro and macro-averaging F-scores for hierarchical classification with SVM classifier were 0.67 and 0.74, respectively [28]. We employed a combination of lexicon-based and ML algorithms and found that the MF + TFIDF feature vector, in combination with the SFM feature selection and the MNB classifier, had the best performance concerning the classifying of our subjects’ messages (ACC = 0.89 ± 0.03, AUC = 0.87 ± 0.03, SEN = 0.94 ± 0.04, and SPE = 0.81 ± 0.03).

There are several studies used in SA in non-English languages. Lenivtceva and Kopanitsa developed a pipeline for unstructured allergy anamnesis by Fast Healthcare Interoperability Resources (FHIR) allergyIntolerance resource in the Russian language. This pipeline had several parts from data preprocessing to semantic analysis. The performance of different parts of this pipeline was measured by F-score which for filtering was 0.94, for allergy categorization was 0.90–0.96, and for allergens reactions extraction 0.90 and 0.93, respectively [29]. In another study, the authors developed different embedding models for diagnosis extraction from electronic medical records in Russian. They used MLP, Convolutional neural networks (CNN), and Long short-term memory networks (LSTMs) classifiers. MLP and CNN had a similar results in three pipelines (F-score was 0.93, 0.95, and 0.97 for pipelines 1–3) [30]. In our study, the best-performing model had F1 score = 0.92.

Figures 3 and 4 indicated MNB was the best classifier with mean AUC = 0.84 across FSs and MF + TFIDF + SFM with mean AUC = 0.84 was the best FS method across classifiers. Followed by, AB (mean AUC = 0.82) and MF + TFIDF + SKB (mean AUC = 0.83) were the next best classifier and FS methods, respectively.

In the method of assessment by the evaluator, 500 h were spent to receive the results of the survey for the satisfaction of the subjects and the observance of service recipients’ rights and to record the data obtained from 250 questionnaires; in addition, the same amount of time was spent to set up the service using artificial intelligence and a survey system so that the information could be permanently accessible. The cost of assessment by the evaluator is equal to that of the implementation of artificial intelligence and an online survey system. The results of Figs. 6 and 7 show that the online survey system used to determine the percentage of patients’ satisfaction in different parts of the hospital and the percentage of observance of service recipients’ rights was comparable to the assessment made by the evaluator. The obtained information remains permanently automatically analyzable, and it will always be possible to furnish feedback. Through this study, feedback on the results of patients’ satisfaction and observance of service recipients’ rights was provided online and instantly for each staff and ward in order to improve efficiency. Since our dataset is imbalanced, in future studies is better used several methods for handling imbalanced datasets [31] including down-sampling, up-sampling and data generation. In the future study, we will use additional sample size gather by an online survey system and develop a deep learning model. In this way, we also will classify neutral comments and use the reinforcement model to improve the performance of the model by itself.

## Conclusion

This study demonstrated that the lexicon-based method, in combination with ML classifiers, could classify patients’ comments into positive and negative categories. We also developed an online survey system capable of analyzing patients’ satisfaction in different hospital wards and removing conventional assessments by the evaluator.

## Electronic supplementary material

Below is the link to the electronic supplementary material.

Supplementary Material 1

## Data Availability

All data generated or analyzed during this study are included in this published article [and its supplementary information files].

## Abbreviations

**HCAHPS**: Hospital Consumer Assessment of Healthcare Providers and Systems
**SA**: Sentiment analysis
**ML**: Machine learning
**DL**: Deep learning
**POS**: Part-of-speech
**BOW**: Bag-of-words
**TFIDF**: Term frequency–inverse document frequency
**NLP**: Natural language processing
**MF**: Multi-feature
**SFM**: SelectFromModel
**SKB**: SelectKBest
**SP**: SelectPercentile
**VT**: VarianceThreshold
**VT + SKB**: VarianceThreshold + SelectKBest
**AB**: AdaBoost
**DT**: Decision Tree
**LR**: Logistic Regression
**MLP**: Multilayer Perceptron
**MNB**: Multinomial Naïve Bayes (MNB)
**TP**: True Positive
**TN**: True Negative
**FP**: False Positive
**FN**: False Negative
**AUC**: Area under the receiver operating characteristic curve
**ACC**: Accuracy
**SEN**: Sensitivity
**SPE**: Specificity
**PRC**: Precision-Recall Curves
