# Supplementary material for: Development of a patients’ satisfaction analysis system using machine learning and lexicon-based methods
Source: BMC Health Serv Res. 2023 Mar 23;23:280. doi: 10.1186/s12913-023-09260-7 (PMC10037842; doi:10.1186/s12913-023-09260-7)
Supplement: Supplementary file 1 — Supplementary Material 1 [file 12913_2023_9260_MOESM1_ESM.docx]

**Table S1**: Hyperparameter and other parameters of the Classifiers

| **Methods** | **Classifier Parameters** | **Hyperparameter Optimization** |
| --- | --- | --- |
| Adaptive Boost {Hastie, 2009 #1} | **Base Estimator:** Decision Tree Classifier  **Number of Estimators** (Number of estimators at which boosting is terminated): 50  **Learning Rate:**1  **Algorithm:** [‘SAMME.R’]  **Random State:** Random State instance (random state is the random number generator) | Number of Estimators = [10,20,30,40,50,60,70,80,90,100,110,120,130] Learning Rate: 0.01 to 1, step: 0.1 |
| Decision Tree | **Criterion** (The function to measure the quality of a split)**:** GINI  **Splitter:** Best  **Max depth**: Nodes are expanded until all leaves are pure  **Min samples split**: 2  **Min samples leaf**: 1  **Min weight fraction leaf:** 0.0  **Max features:** Consider max features at each split  **Random state:** True  **Max leaf nodes:** Unlimited number of leaf nodes  **Min impurity decrease:** 0.0  **Class weight:** one for each class  **Presort** **:** False | Criterion: ['gini', 'entropy']  Max depth: [6, 8, 10, 12, 14, 16, 18, 20, 22, 24] |
| Logistic Regression | **Penalty**: L2  **TOL** (Tolerance for stopping criteria): 1e-4  **C** (Inverse of regularization strength): 1  **Fit intercept**: True  **Intercept scaling** (he synthetic feature weight is subject to l1/l2 regularization as all other features)**:** 1  **Class weight**: balanced  **Random state:** RandomState instance (random state is the random number generator)  **Solver**: ’liblinear’  **Max iter :** 100  **Warm start** (fit a whole new ensemble) **:** False | Solver =['newton-cg', 'lbfgs', 'liblinear'] |
| Multilayer Perceptron | **Hidden layer sizes:** (100,)  **Activation**: Tanh  **Solver** (solver for weight optimization.)**:** Adam  **alpha** (L2 penalty “regularization term" parameter)**:** 0.0001  **Batch size:** Number of samples  **Learning rate:** adaptive  L**earning rate init:** 0.001  **Max iterations** (maximum number of iterations)**:** 1000  **Shuffle**: True  **Momentum:** 0.9  **TOL** (Tolerance for stopping criteria)**:** 1e-4  **Epsilon:** 1e-8 | Hidden layer sizes: [50, 100, 200]  Activation: ['identity', 'logistic', 'tanh', 'relu']  Solver: ['lbfgs', 'sgd', 'adam'] |
| Multinomial Naïve Bayesian | **Priors** (prior probability of each class)**:** According to data distribution | - |
